# Barriers to Pre-Exposure Prophylaxis (PrEP) use for HIV: an integrative review

**DOI:** 10.1590/0034-7167-2021-0963

**Published:** 2023-06-26

**Authors:** Marcela Antonini, Ingred Evangelista da Silva, Henrique Ciabotti Elias, Larissa Gerin, Aliete Cunha Oliveira, Renata Karina Reis

**Affiliations:** I Universidade de São Paulo. Ribeirão Preto, São Paulo, Brasil; II Escola Superior de Enfermagem de Coimbra. Coimbra, Portugal

**Keywords:** Pre-Exposure Prophylaxis, Anti-HIV Agents, Prevention and Control, Access to Health Services, Sexual Health, Profilaxis Pre-Exposición, Fármacos Anti-VIH, Prevención de Enfermedades, Barreras de Acceso a los Servicios de Salud, Salud Sexual, Profilaxia Pré-Exposição, Fármacos Anti-HIV, Prevenção de Doenças, Barreiras ao Acesso aos Cuidados de Saúde, Saúde Sexual

## Abstract

**Objectives:**

to identify and synthesize scientific evidence on the barriers and difficulties for Pre-exposure Prophylaxis (PrEP) use and compliance for HIV.

**Methods:**

an integrative literature review, using the MEDLINE/PubMed, Cumulative Index to Nursing and Allied Health Literature (CINAHL), Academic Search Premier and Scopus (Elsevier) databases.

**Results:**

all (100%) the articles included identified that PrEP users experience some type of structural barrier related to health services such as long distance from the units, suboptimal logistics for taking pills and professional resistance to prescribing PrEP. Furthermore, 63.21% identified social barriers, such as stigma about sexuality and HIV, in addition to individual barriers such as alcohol use, adverse effects, and concerns about long-term toxicity.

**Conclusions:**

the barriers to PrEP use are multifactorial. Effective interventions are needed to support PrEP users in accessing, complying with, and retaining health services.

## RESUMO

**Objetivos:**

identificar e sintetizar as evidências científicas sobre as barreiras e dificuldades para o uso e adesão da Profilaxia Pré-exposição (PrEP) para o HIV.

**Métodos:**

revisão integrativa da literatura, utilizando as bases de dados MEDLINE/PubMed, *Cumulative Index to Nursing and Allied Health Literature* (CINAHL), *Academic Search Premier* e Scopus (Elsevier).

**Resultados:**

todos (100%) os artigos incluídos identificaram que os usuários da PrEP experimentam algum tipo de barreira estrutural relacionada aos serviços de saúde, como longa distância das unidades, logística subótima para retirada de pílulas e resistência profissional para prescrição da PrEP. Ademais, 63,21% identificaram barreiras sociais, como estigma sobre a sexualidade e HIV, além de barreiras individuais, como uso de álcool, efeitos adversos e preocupações com a toxicidade a longo prazo.

**Conclusões:**

multifatoriais são as barreiras para o uso da PrEP. Intervenções efetivas são necessárias para apoiar os usuários da PrEP no acesso, adesão e retenção nos serviços de saúde.

## RESUMEN

**Objetivos:**

identificar y sintetizar evidencias científicas sobre las barreras y dificultades para el uso y la adherencia a la Profilaxis Pre-Exposición (PrEP) para el VIH.

**Métodos:**

revisión integrativa de la literatura, utilizando las bases de datos MEDLINE/PubMed, Cumulative Index to Nursing and Allied Health Literature (CINAHL), Academic Search Premier y Scopus (Elsevier).

**Resultados:**

todos (100%) de los artículos incluidos identificaron que los usuarios de PrEP experimentan algún tipo de barrera estructural relacionada con los servicios de salud, como la larga distancia de las unidades, la logística subóptima para la toma de pastillas y la resistencia profesional a prescribir la PrEP. Además, el 63,21% identificó barreras sociales, como el estigma sobre la sexualidad y el VIH, además de las barreras individuales como el consumo de alcohol, los efectos adversos y las preocupaciones sobre la toxicidad a largo plazo.

**Conclusiones:**

las barreras para el uso de la PrEP son multifactoriales. Se necesitan intervenciones eficaces para ayudar a los usuarios de la PrEP a acceder, adherirse y conservar los servicios de salud.

## INTRODUCTION

Human immunodeficiency virus (HIV) infection remains a public health problem worldwide. Since the onset of the pandemic, 79.3 million people have become infected with HIV and 36.3 million have died from AIDS-related illnesses worldwide. In 2020 specifically, 1.5 million people were diagnosed and 680,000 died from AIDS^(1)^.

In recent years, new HIV prevention strategies have emerged, with Pre-Exposure Prophylaxis (PrEP) considered one of the most important recent biomedical advances in HIV prevention. The combination pill regimen containing oral emtricitabine/tenofovir disoproxil fumarate (FTC/TDF) has been proven to be a highly effective prevention strategy for women and men, with HIV protection greater than 90% among those with high rates of medication compliance^(2)^.

PrEP is indicated for HIV-seronegative people who are at greater risk of becoming infected due to specific vulnerabilities and social contexts and has been implemented worldwide by public health policies aimed at HIV prevention. The World Health Organization (WHO) and Joint UNAIDS have made PrEP implementation a priority for populations most at risk, and several countries have developed national guidelines and plans for its implementation^(3)^. Brazil was the first country in Latin America to use this prevention strategy that took place in the Unified Health System (SUS - *Sistema Único de Saúde*) in 2017 among population segments that concentrate the highest prevalence of HIV in the country, such as gay men and other men who have sex with men (MSM), transgender people, sex workers and HIV-positive couples^(4)^.

Since the implementation of PrEP in the country, there are 473 dispensing services and 64,066 thousand people have benefited from prophylaxis use, 24,843 thousand have discontinued its use and currently there are approximately 39,223 thousand active PrEP users. Thus, it is observed that 39% of people who started PreP discontinued prophylaxis use at some point^(5)^. Although the effectiveness is well established in the literature, compliance and retention of individuals using PrEP in reference services is a challenge. A study carried out in San Francisco in the United States showed that less than half of people who started PrEP were retained in clinical services^(6)^.

In the literature, little is known about the barriers and specific facilitators for PrEP compliance, particularly in some more vulnerable populations such as young black people from sexual and gender minorities in a real environment of PrEP use^(7)^ and further studies are needed to survey detailed of these gaps.

Although PrEP is a highly effective prevention technology, if taken consistently, compliance is therefore critical to the success of the method, and because of this, early discontinuity and gaps in use limit the potential impact of the strategy^(8)^. Multiple approaches are needed to understand and address the complex challenges of PrEP implementation^(9)^. In this regard, it is necessary that health professionals who are aware of this challenge can support their clients with information at the beginning, as well as support them during PrEP use with personalized compliance strategies^(9)^, to mitigate possible barriers to prophylaxis use and compliance

Given this scenario, by understanding the relevance of the strategy in the context of combined prevention of HIV infection and in view of official data from the Brazilian government on the high rate of PrEP discontinuity, we proposed this study in order to gather the currently available evidence on the barriers related to PrEP use. We hope this review can contribute to evidence-based care practice for providing PrEP in real life.

## OBJECTIVES

To identify and synthesize scientific evidence on the barriers and difficulties in PrEP use and compliance for HIV.

## METHODS

This is an integrative literature review that followed the following steps: theme identification and research question elaboration, establishment of inclusion/exclusion criteria, literature search, data extraction and categorization, critical analysis of selected publications, interpretation of results and presentation/synthesis of knowledge^(10-11)^.

The guiding question was constructed from the acronym PIO^(12)^. Thus, it was established: the “population” (P) = PrEP users; the “intervention” (I) = PrEP use and the “outcome” (O) = barriers and difficulties for PrEP use, compliance and continuity. The research question, therefore, was: what are the barriers and difficulties that PrEP users experience in complying with and continuing prophylaxis use?

The search for studies took place in February 2021 in four databases, namely: MEDLINE/PubMed, Cumulative Index to Nursing and Allied Health Literature (CINAHL), Academic Search Premier and Scopus (Elsevier). The search strategy consisted of descriptors and their synonyms identified in Health Science Descriptors (DeCS), Medical Subject Headings (MeSH) and CINAHL headings. The descriptors used were PrEP, HIV, compliance with treatment and access to health services. Bearing in mind that PrEP is a recent strategy, we used filters to limit the search for files from the year 2000 onwards in some databases.

We included primary studies that aimed to assess the barriers, or difficulties, or experiences and challenges regarding PrEP use reported by people who were using prophylaxis or who had already used it at some point in their lives. There were no language restrictions on including files.

In addition to studies that did not meet the inclusion criteria, gray literature, studies that assessed barriers to PrEP use among clinical trial participants or that PrEP delivery did not occur in a real environment were excluded. Studies with mixed participants (participants who used PrEP and participants who never used it) were excluded due to the impossibility of identifying which barriers were experienced specifically by those who used PrEP.

We used the Rayyan platform^(13)^ for screening the studies and for extracting the data we used a script^(14)^ that proposes the main data to be considered in the publication: authorship, country where the study was carried out, journal title, study design (experimental, quasi-experimental, observational, studies with a qualitative approach or mixed methods) and the level of evidence that was classified into five levels^(14-15)^.

Data were also presented regarding the study population, objective or research question and main results found. To analyze the assessment of studies’ level of evidence, we considered: level 1 - meta-analysis of randomized and controlled clinical trials; level II - randomized and controlled clinical trials; level III - clinical trials without randomization; level IV - case-control and cohort studies; level V - systematic reviews; descriptive and qualitative studies; level VI - opinions of authorities and/or opinion of expert committees^(15-16)^.

To describe the identified barriers, we adopted thematic analysis which is a method to identify, analyze and report patterns (themes) within the data^(17)^. Barriers and difficulties for PrEP use were grouped by similarity into three aspects: individual barriers - which include those that are behavioral or clinical and are generally related to a person’s decision-making, attitudes or perspectives; social and interpersonal barriers - defined as those that may derive from the social context in which an individual is inserted; and structural and logistical barriers that are beyond an individual’s control, include barriers to accessing health services and policies, and involve aspects related to institutions and the environment^(18-19)^.

## RESULTS

**Figure 1:**
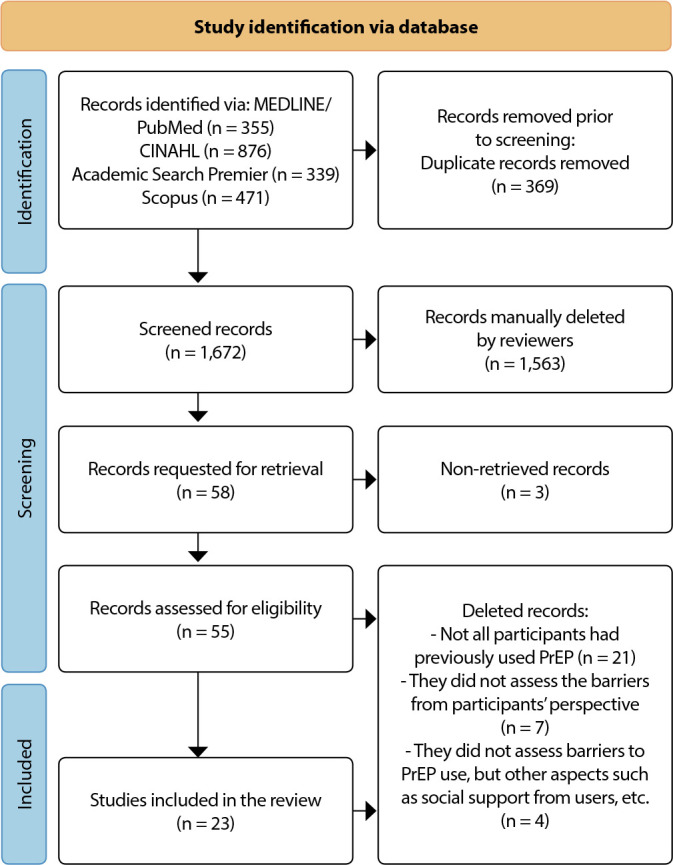
PRISMA flowchart of the research process, study identification and literature selection, Ribeirão Preto, São Paulo, Brazil, 2022

The search resulted in 2,041 files that were exported to the Rayyan platform^(13)^ where it went through the duplicate exclusion process (n = 183). Then, 1,672 articles were evaluated by title and abstract by two independent reviewers. At this stage, 1,563 articles were excluded, leaving 109 files. A third independent reviewer resolved 68 conflicts, resulting in 58 files being read in full. Of these, 03 files were not rescued. Finally, 55 files were read in full and 23 were included in this review, as shown in Figure 1.

Of the 23 (100%) studies included in this review, fifteen (65.2%) were qualitative, five (21.73%), quantitative, and three (13.04%), mixed methods. Of the total, 16 (69.56%) were performed in the United States^(7,18-32)^, five (21.73%), in countries on the African continent^(33-37)^, one (4.34%), in Germany^(38)^, and one (4.34%), in Mexico^(39)^.

Data categorization by similarity consisted of the following groupings and number of articles: individual barriers (15), social barriers (15) and structural barriers (22). All (100%)^(7,18-39)^ studies identified that PrEP users experienced more than one barrier to its use, compliance or continuity and, therefore, some studies were allocated into two or more categories of this review, as shown in Chart 2.

**Chart 1:** Search strategy and databases, Ribeirão Preto, São Paulo, Brazil, 2022

Database	Search strategy
MEDLINE /PubMed	#1 (pre-exposure prophylaxis OR “PrEP” OR “Pre Exposure Prophylaxis” OR “Pre-Exposure Prophylaxi” OR “Prophylaxis, Pre-Exposure” OR “Pre-Exposure Prophylaxis (PrEP)” OR “Pre Exposure Prophylaxis (PrEP)” OR “Pre-Exposure Prophylaxi (PrEP)” OR “Anti-HIV Agents” OR “Agents, Anti-HIV” OR “Anti HIV Agents” OR “Anti-AIDS Agents” OR “Agents, Anti-AIDS” OR “Anti AIDS Agents” OR “Anti-HIV Drugs” OR “Anti HIV Drugs” OR “Drugs, Anti-HIV” OR “AIDS Drugs” OR “Drugs, AIDS” OR “Anti-AIDS Drugs” OR “Anti AIDS Drugs”)
	#2 (HIV Infection [MeSH] OR HIV [MeSH] OR “Human Immunodeficiency Virus” OR “Immunodeficiency Virus, Human” OR “Immunodeficiency Viruses, Human” OR “Virus, Human Immunodeficiency” OR “Viruses, Human Immunodeficiency” OR “Human Immunodeficiency Viruses” OR “Human T Cell Lymphotropic Virus Type III” OR “Human T-Cell Lymphotropic Virus Type III” OR “Human T-Cell Leukemia Virus Type III” OR “AIDS Virus” OR “AIDS Viruses” OR “Virus, AIDS” OR “Viruses, AIDS”)
	#3 (medication adherence [MeSH] OR treatment adherence and compliance [MeSH] OR “Drug Adherence” OR “Adherence, Drug” OR “Medication Nonadherence” OR “Nonadherence, Medication” OR “Medication Noncompliance” OR “Noncompliance, Medication” OR “Medication Non-Adherence” OR “Medication Non Adherence” OR “Non-Adherence, Medication” OR “Medication Persistence” OR “Persistence, Medication” OR “Medication Compliance” OR “Compliance, Medication” OR “Medication Non-Compliance” OR “Medication Non Compliance” OR “Non-Compliance, Medication” OR “Drug Compliance” OR “Compliance, Drug” OR “Therapeutic Adherence and Compliance” OR “Treatment Adherence” OR “Adherence, Treatment” OR “Therapeutic Adherence” OR “Adherence, Therapeutic”)
	#4 (health services accessibility [MeSH] OR “Health Services Availability” OR “Accessibility of Health Services” OR “Accessibility, Health Services” OR “Access to Health Services” OR “Access to Health Care” OR “Access to Therapy” OR “Access to Therapies” OR “Therapy, Access to” OR “Access to Treatment” OR “Access to Treatments” OR “Treatment, Access to” OR “Health Services Geographic Accessibility” OR “Program Accessibility” OR “Accessibility, Program” OR “Access To Medicines” OR “Access To Medicine” OR “Access to Medications” OR “Access to Medication” OR “Medication, Access to” OR “Medication Access” OR “Access, Medication”))
	#1 AND #2 AND #3 AND #4
CINAHL	(pre-exposure prophylaxis or prep or preexposure prophylaxis) OR HIV pre exposure prophylaxis AND (healthcare or health care or hospital or health services or health facilities) AND (barriers or obstacles or challenges) AND (patient compliance or patient adherence)
Academic Search Premier	(pre-exposure prophylaxis or prep or preexposure prophylaxis) OR hiv pre exposure prophylaxis AND ( healthcare or health care or hospital or health services or health facilities ) AND ( barriers or obstacles or challenges ) AND ( patient compliance or patient adherence )
SCOPUS	(TITLE-ABS-KEY(“pre-exposure prophylaxis”) AND TITLE-ABS-KEY (“HIV”) AND TITLE-ABS-KEY (“Health Services Accessibility”) OR TITLE-ABS-KEY (“medication adherence”) OR TITLE-ABS-KEY (“treatment compliance”)) AND (LIMIT-TO(PUBYEAR, 2021) OR LIMIT-TO PUBYEAR, 2020) OR LIMIT-TO (PUBYEAR, 2019) OR LIMIT-TO (PUBYEAR, 2018) OR LIMIT-TO (PUBYEAR, 2017) OR LIMIT-TO (PUBYEAR, 2016) OR LIMIT-TO (PUBYEAR, 2015) OR LIMIT-TO (PUBYEAR, 2014) OR LIMIT-TO (PUBYEAR, 2013) OR LIMIT-TO (PUBYEAR, 2012) OR LIMIT-TO (PUBYEAR, 2011)) AND ( LIMIT-TO(DOCTYPE, “ar”))

**Chart 2:** Synthesis of studies included in the review according to year of publication and level of evidence, Ribeirão Preto, São Paulo, Brazil, 2022

Authors, year and country of study	Objective of study	Population/sample studied	Method/strategy used	Main findings	LoE^*^
Arnold et al., 2017^(18)^/ USA	Explore the structural, social, behavioral and clinical factors that affect PrEP use and retention in care.	Young MSM (n=30)	Qualitative/individual interviews	The main factors that affect PrEP use and retention in PrEP care include structural factors (cost, assistance with doctor visits and drug payments), HIV stigma and relationship status, partner’s HIV status, risky sexual behaviors and clinical factors (side effects).	V
Ellison et al., 2019^(20)^/ USA	Assess barriers to oral PrEP and influences of sociodemographic characteristics and sexual behavior in choosing new drug formulations.	MSM (n=108)	Quantitative, cross-sectional/individual interviews	The listed barriers were daily PrEP regimen, difficult access to PrEP prescribers, difficulty making appointments, picking up prescriptions and talking to health professionals prescribing PrEP, racial and ethnic differences (Black and Hispanic men face more barriers compared to white and Asian men).	IV
Hunt et al., 2019^(21)^/ USA	Examine the challenges of accessing and complying with PrEP and to assess the usefulness of objectively monitoring PrEP compliance via urine.	Young adults (aged 18 - 34) using PrEP (n=40)	Quantitative, cross-sectional, descriptive	Participants reported being able to access PrEP quickly, but notable barriers reported included the unwillingness of the provider to prescribe PrEP. Regarding compliance, the most frequent barrier was remembering to take the medication.	V
Park et al., 2019^(22)^/ USA	Characterize the path to PrEP gathering and continuity.	Black or Latina heterosexual cisgender women (n=14)	Qualitative	Misinformation about PrEP among healthcare professionals, concerns about medication safety, difficulty filling and withdrawing PrEP at pharmacies, out-of-pocket health costs.	V
Rice et al., 2019^(23)^/ USA	Examine perceptions of access to PrEP.	Men who use PrEP (n=44)	Semi-structured individual interviews	The main barriers were cultural and social norms that silence the discussion about sexuality, lack of awareness and dissemination about PrEP, stigma related to sexuality, concerns about the adequacy and technical quality of PrEP services. The structural ones were long distances from health services, conflicting appointment times, medication costs, lack of health insurance resources, lack of knowledge of PrEP by health professionals, low perceived risk of HIV, concern about side effects.	V
Schwartz et al., 2019^(24)^/ USA	Better understand the experiences of MSM in the adoption of PrEP.	MSM (n=38)	Qualitative/individual interviews	Stigma about PrEP from the very health professionals who provide PrEP, stigma of health professionals about homosexuality, social stigma about PrEP use being related to promiscuity, perception of discomfort of health professionals in talking about sexual health with gay men.	V
Sun et al., 2019^(25)^/ USA	Identify barriers and facilitators to accessing PrEP.	Gay and cisgender men (n=27)	Qualitative	Long distances to access PrEP, living in a rural area, difficulty connecting to a PrEP prescriber, costs to buy the drug and interruptions in the supply of PrEP in pharmacies, long distances to access PrEP, living in a rural area, difficulty connecting to a professional PrEP prescriber, costs to purchase the drug, and interruptions in the supply of PrEP at pharmacies.	V
Wood et al., 2019^(7)^/ USA	Identify barriers and facilitators to HIV PrEP compliance.	MSM and trans women (n=31)	Nested mixed method with a prospective cohort	Stigma related to being mistaken for someone with a positive HIV status, HIV-related homophobia, stigma related to skin color, fear of having one’s sexuality revealed to one’s family, inaccessibility of health systems, side effects, stressors related to routine life and low perception of HIV risk.	IV
Laborde et al., 2020^(26)^/ USA	Interpret the difficulties in PrEP persistence in a context of structural barriers, as well as clinical, pharmaceutical and interpersonal experiences.	Adult PrEP users (n=25) and health professionals prescribing PrEP (n=18)	Qualitative/individual interviews	Sexuality-related stigma, medical distrust, difficulty meeting PrEP follow-up requirements such as scheduling and attending routine appointments and laboratory tests, housing instability, substance use, mental health, difficulty communicating with providers. Getting refills from pharmacies was inconvenient and alienating for some participants.	V
Nieto et al., 2020^(27)^/ USA	Explore reasons for discontinuing PrEP.	Black and Latino MSM and Black and Latino transgender women (n=22)	Qualitative/interviews with a semi-structured script	Lower perceived risk of HIV related to changes in sexual behavior, loss or change of health insurance, long distances to health services, difficulty attending routine appointments, anticipated and experienced adverse medication effects, fear of PrEP interaction with hormones or overloading the body with too many medications.	V
Nieto et al., 2020^(28)^/ USA	Identify barriers and drivers for PrEP adoption.	Black and Latino trans women (n=18)	Qualitative/individual semi-structured interviews	Structural, logistical, linguistic, and cultural barriers to physician engagement, lack of competent transgender care, and prioritization of hormone therapy over PrEP use.	
Owens et al., 2020^(29)^/ USA	Raise barriers and facilitators to PrEP use.	MSM and who live in rural areas (n=34)	Quantitative/semi-structured interviews	Lack of rural dissemination of information about PrEP, health professional not informing about PrEP, concern about medication side and adverse effects, cost of PrEP compliance and involvement, lack of access to PrEP care and quality of care, especially in a rural environment.	IV
Camp & Saberi, 2021^(30)^/ USA	Understand enablers and barriers to on-demand PrEP use and preferences for PrEP regimens, challenges to PrEP use during the pandemic.	MSM (n=140)	Cross-sectional, quantitative, with online data collection	Unplanned sexual encounters, difficulty remembering the dose, unpreparedness of the professional to provide PrEP care, the COVID-19 pandemic (difficulty in performing/obtaining laboratory tests, having PrEP refills, receiving a refill prescription from a healthcare professional, not being able to get a healthcare professional consultation, and not being able to communicate with your healthcare professional).	V
Felsher et al., 2021^(31)^/ USA	Describe the context of lives of people who use drugs and the challenges of compliance with PrEP.	Cisgender women who inject drugs (n=23)	Qualitative/individual interviews with semi-structured script	Difficulty meeting basic survival needs arising from the intersection of opioid use disorder, economic insecurity and housing instability, forgetting to take medication regularly, lack of secure storage of pills, loss or theft of pills, low self-efficacy, disabling structural factors (prescription drug market, drug treatment facility, or prison facility).	V
Jackson-Gibson et al., 2021^(33)^/ Kenya	Explore enablers and barriers to PrEP implementation, acceptance and persistence.	Adolescent girls and young women (n=40)	Qualitative/individual interviews and focus groups	Side effects (lack of appetite, dizziness, nausea, vomiting, and stomach pains), PrEP-related community stigma, geographic distance to health facility, PrEP use associated with increased promiscuity, commercial sex workers, and people infected with PrEP HIV.	V
Kadiamada-Ibarra et al., 2021^(39)^/ Mexico	Identify barriers and facilitators of a PrEP compliance program.	Male sex workers (n=8)	Quali-quantitative/individual interviews + focus groups	Lack of properly focused strategies to reach male sex workers, stigma related to HIV, PrEP use and key populations, geographic distance from PrEP facilities, lack of information about where to get PrEP and costs, lack of public policy, limitation of PrEP provision at ImPrEP sites and in implementation studies.	V
Kimani et al., 2021^(34)^/ Kenya	Explore reasons for PrEP persistence.	Transgender women and men who have sex with men (n=53)	Mixed study/qualitative stage with semi-structured interview	Daily dosing schedule was a nuisance. Withdrawal of 90-day pills, negative reactions from partners and stigmatizing health services were considered barriers.	V
Koppe et al., 2021^(38)^/ Germany	Analyze factors associated with stopping PrEP, barriers that may prevent people from continuing PrEP, and investigate sexual behavior after stopping PrEP.	Adults who used or were using PrEP (n=4,848)	Cross-sectional, quantitative/online data collection	The barriers described are often modifiable. Barriers were referred to as changing sexual partners, satisfaction with other prevention strategies, lower perception of risk of acquiring HIV, side effects, not wanting to take a chemical substance, fear of long-term side effects. Logistical barriers were difficulty obtaining PrEP, difficulty finding a doctor who prescribes PrEP, financial burden, and lack of health insurance coverage.	V
Ogunbajo et al. 2021^(19)^/ USA	Understand barriers to compliance experienced for PrEP use.	Black and Hispanic/Latino trans women and non-binary individuals (n=30)	Qualitative/individual interview	Concerns about costs, mental health issues, substance use, and concerns about PrEP side effects, including hormone interaction, intimate/romantic partner influence, and the impact of patient-professional communication, stigma, and negative community views related to PrEP, negative experiences in healthcare settings, unreliable transportation, employment, and housing insecurity.	V
O’Rourke et al., 2021^(35)^/ Cape Town	Explore PrEP use experiences, including compliance and persistence or discontinuation experiences.	Adolescent girls and young women (n=22)	Qualitative prospective cohort	Pill-taking challenges, social opposition, or traumatic/unexpected event. Feelings of disappointment/failure regarding inability to continue PrEP use, PrEP-related stigma, decreased motivation, cultural issues such as conflict with coming-of-age traditions, lack of family support.	V
Sack et al., 2021^(36)^/ Mozambique	Explore the perspectives, attitudes and experiences of HIV-serodiscordant partners taking PrEP.	People in HIV-serodiscordant relationships (n=19)	Qualitative/individual semi-structured interviews	The factors that influence PrEP compliance were divided into individual, interpersonal and organizational. Individual factors were love for the partner, knowledge about PrEP and belief that the drug is effective, and fear of HIV and PrEP stigma. Interpersonal factors affecting PrEP uptake were desire to protect family, partner support and relationship strength, overcoming fear of stigma to seek support from family and friends, and gender compliance approaches.	V
Stoner et al., 2021^(37)^/ Cape Town	Understand patterns of decline in PrEP compliance in a prospective longitudinal cohort study.	Adolescent girls and young women (n=22)	Qualitative prospective cohort	Lack of family or partner support, traumatic events, and changes in the partnership. Decreased motivation, logistical barriers related to the health service, difficulty with the PrEP routine, forgetfulness, being busy, getting sick, changing residence, taking care of children, participating in cultural activities and difficulty in attending clinical appointments.	IV
Willie et al., 2021^(32)^/ USA	Understand the multilevel factors that influence PrEP persistence.	Black cisgender women (n=8)	Qualitative/ individual interviews	Accessibility and costs of PrEP services, medication side effects (upset stomach, constipation, tachycardia and morning sickness).	V

* *LoE - level of evidence.*

### Individual barriers

The identified individual barriers were related to the suboptimal intake of PrEP related to the difficulty of using medications orally^(26-27,35)^, forgetting to take the medication daily^(21)^, lack of safe storage of pills^(31)^ and not wanting to take a chemical substance for a long time^(26,38)^. Moreover, alcoholic beverage and other drug consumption^(19,31)^, lifestyle and competing stressors that involve the need to reconcile personal life and work routine with PrEP use also directly interfered with compliance^(7,19,21,35,37)^.

Adverse effects of medication were identified as a barrier to compliance. Most participants who experienced adverse effects reported a transient onset of headaches and nausea that resolved within a few days of using PrEP^(7,35,38)^. Other reported effects, such as lack of appetite, dizziness, vomiting and stomach pains were reasons for discontinuing and abandoning prophylaxis^(26,33,38)^, especially when these effects involved high levels of creatinine^(27-28)^.

Concerns about future PrEP side effects, long-term drug toxicity^(7,18,23,27-28,38)^ and hormone interaction among transgender people was cited as a reason for discontinuing prophylaxis. Three studies identified that transgender women on hormone therapy expressed fear of interaction of PrEP with hormones and fear of a harmful overload of drugs in the body^(19,27-28)^.

Low perceived vulnerability to HIV resulted in sporadic use, suboptimal compliance, discontinuity of PrEP, or drug use only on weekends or in periods of changes in sexual behavior^(7,23,27-28,35,38)^. Furthermore, relationship status leading to partner belief and trust has been reported as barrier to PrEP use in long-term monogamous relationships^(18)^.

The presence of mental health-related problems (e.g., depression, anxiety) are challenges that affect motivation for sustained compliance with prophylaxis, making it less of a priority^(19,22)^. However, sustained psychoactive substance use, especially opioid drugs, is an important barrier.

In contexts of drug use associated with economic insecurity and housing instability, meeting basic survival needs becomes a priority that overshadow the regular prophylaxis use and therefore is a barrier to its compliance^(19,31)^. The daily movement of meeting basic human needs such as food, hygiene and housing, and looking for ways to take psychoactive substances were reported as a priority, leaving the dedication to using PrEP at a level far from being a priority^(31)^.

Barriers have been described among individuals using prophylaxis known as “on-demand”, “episodic”, “event-based” or “event-oriented” PrEP and were linked to unplanned sexual encounters, resulting in missing a PrEP dose before sexual activity and difficulty remembering the dose after a double dose of the drug^(30)^.

In contrast, the daily PrEP regimen was reported to be challenging in some studies^(20,34)^, which is why many users expressed a desire to have long-acting formulations. Replenishing PrEP for 90 days was raised as discouraging by users who felt overwhelmed, with the feeling of having a long way to go, which resulted in feeling powerless to deal with the regular use of all those pills for a long period of time^(34,37)^.

### Social and interpersonal barriers

Despite the numerous individual barriers raised, PrEP use and continuity was more impacted by external challenges, making discontinuity unintentional in several studies. Interpersonal barriers included the influence of intimate/romantic partners on the ability to initiate and keep using, as partners were hesitant about using PrEP which was perceived as a lack of trust and loyalty within the relationship^(18-19)^. The partner’s motivation for the use and persistence in PrEP was reported as a key factor for compliance, and the decrease or disapproval of prophylaxis use by them led to PrEP discontinuity by many participants^(19,35)^.

Regarding social barriers, stigma was a barrier cited for compliance in different studies and is described in multiple ways^(7,18-20,23-28,31,33-36,38)^. A stigma on PrEP use was identified, based on community’s negative perceptions that mistakenly believed that prophylaxis was linked to sexual behaviors such as promiscuity^(7,18-20,23-28,31,33-36,39)^.

HIV-related stigma is also a prominent barrier to accessing PrEP and health services^(7,18,33,36,39)^. Stigma was felt at the individual level, related to the fear of knowing their HIV status, which prevents individuals from seeking out preventive services, and at the societal level, with concern of the potential stigma they face by community members and other male sex workers if they were seen receiving HIV-related services^(39)^ who associate PreP use with HIV treatment due to similar pill appearance and packaging^(33)^.

It is important to emphasize that, in addition to these social pressures, the fear of having one’s sexuality revealed to family members or in the community^(7,39)^, in addition to the fear of having the serological status of HIV-positive partners revealed^(36)^, were relevant barriers listed by several PrEP users.

Another interesting aspect concerns the social norms that silence the discussion about sexuality and favor the judgment about sexual orientation as potential barriers^(23)^. In more than one of the studies conducted on the African continent, PrEP users discontinued prophylaxis due to cultural traditions^(35,37)^. In this case, the rituals to become a traditional healer in the community generated an identity conflict for users who chose to discontinue prophylaxis for fear of interfering with initiation rituals of this cultural activity^(35)^.

Another barrier found was experiencing traumatic events, such as grief. Dealing with the physical loss of family members and close people, including those involved in emotional support for PrEP use, was a precursor to discontinuing prophylaxis^(35)^.

### Structural barriers

Structural barriers, for the most part, were related to prolonged waiting time for a consultation with prescribers and difficulties in communicating with these professionals, administrative delays in the health system, lack of medication in some health units and medication cost (unlike Brazil, in some countries, PrEP is not free), ignorance and/or resistance of some health professionals to prescribing PrEP, health team stigma and difficulty in accessing health services^(7,18-39)^.

Lack of education about PrEP is a major barrier to PrEP uptake, particularly among highly vulnerable populations^(39)^. Misinformation from health professionals with inaccurate or limited information^(22-23)^, insufficient number of qualified health professionals to offer PrEP^(23,28-29,33,38)^ and the perceived stigma coming from the health professionals themselves^(7,20-21,23-24)^ remain a challenge in the current implementation strategy, making it difficult to initiate, comply with and maintain prophylaxis.

Lack of competent medical care for transgender people^(28)^, professional unpreparedness to deal with gender diversity, sexual identity and sexual behavior as well as with the clinical management of PrEP^(19-25,29-30)^ were raised in several articles such as a significant barrier to PrEP. In fact, one of the studies identified that this context provided users with uncomfortable and sometimes negative experiences with health professionals, who were described as culturally insensitive or lacking knowledge about transgender people’s health^(19)^.

Costs related to PrEP as well as ancillary health services (i.e. doctor visits and laboratory tests) and lack of health insurance coverage were barriers to both acceptance and compliance^(19,23,25,29)^. Other aspects identified were loss of health insurance after moving to a new city or employment, resulting in higher costs to continue with a PrEP prescription^(18,25,27-28)^ and difficulty in keeping up with follow-up requirements, with consultations laboratory tests^(23,26)^. Limited financial resources in health facilities also make it difficult to initiate and clinically monitor side effects. This included inability or delays in required laboratory tests resulting in skipping tests to avoid delay in starting PrEP^(23)^.

However, long distances traveled to reach health services, difficulty in renewing prescriptions, picking up medication or attending routine follow-up medical appointments, in addition to long waiting times for pharmacies to restock, they were also reported as barriers to PrEP in several articles^(18,22-23,25,27-29)^.

Even living in a rural area was reported as a significant barrier, not only because of the long distance from health services, but also because of the lack of dissemination of prophylaxis among the rural community, even when it is in contact with health professionals. This made the population aware of PrEP through friends and social networks and resulted in the need to research by themselves to request the prescription from health professionals^(29)^.

Finally, one of the studies identified the impact of the COVID-19 pandemic, which brought important barriers, such as the difficulty in performing/obtaining laboratory tests, obtaining PrEP refills, receiving a refill prescription from a health professional, not having an appointment or not being able to communicate with a health professional^(30)^.

## DISCUSSION

PrEP is a significant advance for HIV prevention, however, this review identified multifaceted barriers and difficulties for its use, compliance and continuity.

There are still many gaps in the identification of these barriers between populations from different regions, since most studies were carried out in the USA^(7,18-32)^ and in countries on the African continent^(33-37)^. No study was observed with the population of Latin American countries, such as Brazil, in which there are social inequalities and inequities that can constitute strong barriers to PrEP use.

Likewise, the need to expand studies focusing on other populations is highlighted, since most were conducted among MSM^(20,24,29-30,34)^ and the transgender population^(7,19,27-28,34)^. These findings may be related to the fact that PrEP implementation has been aimed especially at key populations. However, other population groups who are at risk of HIV infection, such as adolescent girls, young women, sex workers, people who use injecting drugs and are deprived of liberty must be understood from a broader perspective that transcends just the MSM population group. The importance of each of these populations varies in the regions of certain countries^(3)^. Furthermore, among MSM there are even more socially marginalized groups such as black MSM who are disproportionately affected by HIV and are therefore considered a priority for PrEP.

Our findings illustrate that the use of this preventive measure is permeated by multiple barriers. All studies identified that PrEP users experienced more than one barrier to PrEP use^(7,18-39)^, and this data is worrisome, since efficacy is directly related to compliance.

Many barriers are modifiable for the same person over time. A study by Koppe *et al*.^(38)^ showed that short-term PrEP users were more likely to discontinue prophylaxis because of concerns about long-term side effects and not wanting to take a chemical substance. In contrast, long-term users more often indicated that their partner status had changed, which was the main reason for discontinuing.

This dynamism is expected because it is a prevention strategy that is directly related to sexual behavior, which can take on different nuances and all this dynamism directly influences the adoption of preventive measures^(40)^. However, it is extremely important to provide conditions to guarantee dignified access and qualified preventive care to the population, respecting their unique moments and contexts of life. In this way, understanding the barriers involved in this prevention movement is essential to outline effective health care strategies for people who seek PrEP.

PrEP is a preventive strategy used by HIV-negative people rather than for treatment purposes, so there can be unique challenges in motivating compliance, including the inconvenience of taking the pill daily, which can be considered one of the barriers to its use^(20-34)^. Moreover, many people find it difficult to ingest medication, and aspects related to the taste, smell and size of the pill can also influence prophylaxis use^(7,26-28,35)^.

Alternative dosing strategies, such as on-demand PrEP, may be more acceptable and manageable for people who have difficulty complying with a daily dosing schedule^(41)^. The formulation of injectable antiretrovirals, long-lasting subcutaneous implants and extended-release vaginal rings are also being studied to minimize the effects of non-compliance, and represent promising alternative options to oral medications^(42)^. This is important, as compliance with preventive strategies is directly linked to their adequacy and convenience in the context and preference of those who consume them^(43)^.

Furthermore, on-demand PrEP may be of particular relevance for those who have infrequent sexual activity, while daily regimens may fit well for those whose sexual events are frequent. However, the complexity in the instructions for following non-daily regimens may require additional attention, so specific tools and supports are needed to support their continued use^(43)^.

Several studies in this review identified that lifestyle can be a barrier to PrEP use^(7,19,21,35,37)^. Stressors such as changes in daily life, long trips, being away from home^(18,25,27-28)^, exhausting workload were associated with forgetting/difficulty remembering to take pills in addition to attending routine appointments^(7,19,21,35,37)^.

Thus, effective personal strategies that help with medication intake, such as using a telephone alarm clock, notes, pill organizer box, should be advised by the health team and can facilitate medication use^(44)^. Still, there is a need for assistance for users to organize their routine in order to preserve prophylaxis use even when there is a need for travel or changes in routine. In Brazil, PrEP is made available by the SUS, which allows people to continue to have access to free care and medication dispensation even when traveling or changing city/state even within the national territory^(45)^.

Several studies in this review identified that medication-related costs were barriers to continuing PrEP use^(19,23,25,28-29)^. Therefore, it is important to emphasize that the institution of PrEP as a public policy in Brazil contributes to the mitigation of potential barriers related to its use. However, it is necessary to identify and overcome several other barriers that permeate the continuum of PrEP-related care.

Belief about adverse and side effects^(7,17,23,27-28,35,38)^, as well as experiencing them^(7,26,33,35,38)^ were important barriers to medication use and compliance. For some people the adverse effects improved with time of use^(7,35,38)^; however, for others, they were enough to discontinue prophylaxis^(26,33,38)^.

In fact, the literature describes the presence of symptoms such as nausea, flatulence, diarrhea, headache and abdominal pain, which tend to reach a peak frequency in the first month after onset and usually resolve in three months, called “start-up syndrome”, which affects the minority of people on PrEP^(46)^. It turns out that the tolerance of discomfort is different for each individual. Therefore, the health team should advise that in general adverse effects tend to decrease with regular use within the first month of use^(44)^, implementing strategies to manage these effects in order to help in coping without giving up on therapy.

In addition, concern about therapeutic safety, fear of possible long-term side effects (e.g., liver damage)^(7,18,23,27-28,38)^, as well as interactions between PrEP and other medications, including those involved in hormone therapy^(19,27-28)^ have been raised as barriers to continued PrEP use.

Sustained psychoactive substance use, especially opioid drugs, is an important barrier to PrEP use. In contexts of drug use associated with economic insecurity and housing instability, meeting basic survival needs become a priority that overshadow the regular PrEP use and, therefore, are configured as barriers to its use and compliance^(19,31)^. Moreover, there is also the fear of medication interaction with alcohol and psychoactive substances^(7,19)^. Despite the numerous individual barriers raised, the use and continuity of PrEP was more impacted by external challenges, making prophylaxis discontinuity unintentional in several studies^(7,18,24,26,33,35-36,39)^. The social context can constitute a barrier to prophylaxis use. Intriguingly, experiencing traumatic events seemed to take center stage with regard to PrEP use. Experiencing acts of violence and rape motivated women to seek and comply with prophylaxis, which was supported by family members and people from the social stronghold. However, when experiencing the death of family members, it culminated in a decrease in the motivation to use PrEP, resulting in its discontinuation^(35)^.

In this review, almost half of studies identified some type of stigma related to PrEP use as a barrier to its use, with more than half of them referring to stigma about HIV^(7,18-19,23-24,26,33-35,39)^. HIV serodiscordant couples reported constant fear of having their partner’s serology revealed in the family or community^(36)^. In addition, the fear that people using PrEP will be seen as HIV-positive is centered on the stigma of the virus itself^(18,43)^. The history of HIV infection and especially AIDS was marked by a social construction based on discrimination and stigma^(47-48)^ that persist until the present day. Although PrEP users are HIV-seronegative, they are embedded in a web of tensions related to the virus and therefore often experience similar situations (such as perceived stigma) to people living with HIV.

Nevertheless, the stigma associated with promiscuity has been described by PrEP users. Prophylaxis has been perceived as an HIV prevention measure for individuals who wish to have sex without using condoms, for instance, or with multiple partners. Thus, some studies have reported that social identity as a PrEP user is often associated with negative perceptions that wanting sex without a condom can be considered promiscuity^(7,18-20,23-28,31,33-37)^.

Combating social stigma, promoting, respecting and protecting human rights is fundamental for human development and the end of AIDS as a threat to public health. Thus, successful PrEP implementation as a response to tackling HIV must use rights-based approaches and combat widespread and entrenched stigma, discrimination and other human rights violations faced by people living with HIV,as well as by population groups that are at increased risk of infection^(3,49)^. However, the construction of eligibility criteria for prophylaxis use specifically on gender identities and sexual preferences that differ from heteronormative hegemony has reinforced the existing stigma on some population groups and on HIV infection^(49)^, fueling prejudiced beliefs and perceptions that people within heteronormative patterns are distant or exempt from the risk of acquiring HIV and therefore do not fit the criteria for PrEP.

In the US, a study identified that people express less support for funding policies and programs that enable access to PrEP for stigmatized groups (key populations) compared to the general population^(49)^. The authors reinforce that public PrEP campaigns that specifically target key populations run the risk of perpetuating existing stereotypes of promiscuity associated with these groups^(49)^.

Outreach and support campaigns for PrEP with messages that reach the general population, avoiding explicitly naming high-risk groups, help to avoid prejudiced beliefs^(49)^. It is essential that in PrEP programs, health professionals understand how users are perceived within the community and how these perceptions can prevent the adoption of strategies and their personal consequences for PrEP use.

For structural barriers, health systems barriers focus primarily on issues of accessibility to clinical or pharmacy services due to insurance, transportation, or difficulty navigating complex health systems^(7,20,23,25,29,32-33,39)^. Logistical factors related to health services^(7,19-20,22-24,26-30,34,37-38)^ and their professionals^(19-25,29-30)^ raised as barriers to PrEP use in this review raise a paradox in PrEP implementation in health services. Service structural unpreparedness (considering here the professional unpreparedness) in the face of the demand for this strategy is configured against the grain of its innovative character.

Care service quality directly influences gathering and compliance with PrEP^(23,29)^. Hostile consultations with resistance to prescribing prophylaxis^(20-21,30)^, as well as difficulty in communicating with the team, prolonged waiting periods for consultations^(19,26,30)^, were identified in this review and show the professional and institutional unpreparedness for this new preventive strategy.

Professional unpreparedness is at the core not only of handling prophylaxis, but also of caring for transgender people^(19-25,29-30)^. This professional and institutional lack of preparation may be related to the norms of the local community and the moral conviction of its health professionals about sexuality^(23)^. There may be a transfer of responsibility in the choice of strategies by health professionals influenced by biopower, reducing people’s autonomy in choosing the most comfortable preventive measure for their context.

Moreover, stigma and discrimination of a sexual nature influence access to health services. Gender inequalities and criminalization of sex work most often prevent MSM, transvestites and transsexuals from seeking PrEP in health services. Ensuring access for these people would enable better self-rated health in addition to reducing HIV transmission. The shortage of professionals in the propagation of this measure, work overload and lack of training of the team in the care and encouragement of specific groups demonstrated importance in terms of access to PrEP by users^(7,19,27-28,34)^ .

In addition, PrEP has been recommended and prescribed mostly by health professionals who work in the area of infectology, including those involved in the care of people living with HIV. However, it is intended for people who are seronegative for the virus, configuring a “reach paradox” of prophylaxis. Therefore, offering PrEP in non-specialized services to the population living with HIV can make it possible to achieve this strategy^(23,32)^ and, consequently, reduce some structural barriers to its use.

Specific strategies that bring PrEP closer to the population as a whole are important. When carried out dynamically as workshops, group activities, among others, the exchange of knowledge is stimulated. Thus, it is believed that health professionals should carry out their counseling without prejudice about sexuality and/or sexual behavior. Educational actions planned according to individuals’ context are totally possible and relevant with regard to sexually transmitted infection prevention^(23)^.

In addition, simplified testing, standing orders to laboratories, PrEP prescriptions valid for up to 90 days, proactive provision of support and medication compliance counseling are also plausible strategies to start overcoming some structural barriers^(26)^.

During consultations, compliance should be addressed largely in a simple and clear manner. Assessing medication intake reinforces that effectiveness is closely linked to compliance, associate taking with daily routine events, avoiding forgetfulness^(31,37)^, observe the pharmacy data regarding the dispensing of medications and assess adverse management are behaviors that reduce barriers^(49)^.

Allowing professionals from different specialties to start offering and prescribing PrEP, in addition to adopting new strategies that encourage compliance, requires efforts at institutional and governmental levels. Ongoing education and adequate training of health professionals on PrEP^(38,49)^ is crucial to ensure successful implementation of HIV prevention programs.

### Study limitations

The results of this review must be interpreted in light of its limitations. Most studies were conducted with MSM in the USA and therefore the barriers identified in this review may not be applicable to other countries due to differences in culture, belief and health systems. Furthermore, another limitation concerns the four bases used in the search strategy, which may have influenced the results. However, this does not invalidate our findings since our search included multidisciplinary databases, recognized and used worldwide. However, for conducting this study, we sought to carry out a systematic and rigorous approach to the processes of an integrative review, particularly data analysis, which implies the reduction of biases and errors.

### Contributions to nursing, health and public policies

The findings of this review provide evidence regarding the barriers to PrEP use and therefore contribute to the discussion on strategies to prevent and combat the HIV/AIDS epidemic. Gathering information about the barriers and potential for the successful PrEP use contributes to the formulation of public policies, as a support for the implementation of preventive strategies in places with an epidemiological situation of high rates of HIV/AIDS cases, for the elaboration of professional training activities as well as for the elaboration of care protocols in units that have implemented PrEP.

## CONCLUSIONS

The findings of this review show that PrEP users experience multifaceted barriers and difficulties for the use and continuity of prophylaxis. These barriers range from individual aspects such as life habits and fear of prophylaxis pharmacological safety, social aspects, such as stigma related to HIV and promiscuity, even structural aspects, such as failures and difficulties related to health services.

Despite the various individual barriers, the use and continuity of PrEP is more impacted by external challenges that are beyond an individual’s control, namely social and especially structural barriers that make prophylaxis discontinuity unintentional.

Overcoming these barriers requires a variety of approaches to address all of these instances. Institutional and governmental efforts that focus on permanent education and professional training of care providers are essential to overcome structural barriers to prophylaxis use. Additionally, it is necessary to understand and respond to barriers to PrEP use among users and members of their social networks.
